# The Relationship Between Evaluation Methods for Chemotherapy-Induced Peripheral Neuropathy

**DOI:** 10.1038/s41598-019-56969-9

**Published:** 2019-12-30

**Authors:** Yoichiro Yoshida, Atsushi Satoh, Teppei Yamada, Naoya Aisu, Taisuke Matsuoka, Tomoko Koganemaru, Ryuji Kajitani, Taro Munechika, Yoshiko Matsumoto, Hideki Nagano, Akira Komono, Ryohei Sakamoto, Mitsuaki Morimoto, Hisatomi Arima, Suguru Hasegawa

**Affiliations:** 1 Department of Gastroenterological Surgery, Fukuoka University Faculty of Medicine, Fukuoka, Japan; 2 Department of Preventive Medicine and Public Health, Faculty of Medicine, Fukuoka University, Fukuoka, Japan

**Keywords:** Neuropathic pain, Neurotoxicity syndromes

## Abstract

Numbness and pain are currently evaluated using subjective methods such as the visual analogue scale (VAS). PainVision (PV) is an analytical instrument that was designed to quantitatively assess sense perception and nociception in patients. Chemotherapy-induced peripheral neuropathy (CIPN) is one of the most important adverse events that renders prolonged chemotherapy difficult. To assess the features of CIPN, we aimed to compare PV methods with existing methods. A total of 73 patients received oxaliplatin for metastatic colorectal cancer. Registered patients included 37 men and 36 women in the range of 37 to 89 years (median 70). CIPN was evaluated a total of 483 times (median per patient six times). Our study examined the correlation between evaluation methods of CIPN using VAS and PV, respectively. The average VAS (hand), VAS (foot) and PV scores of CIPN were 18.4 (range: 0–100), 23.8 (range: 0–100), and 24.7 (range: 0–496), respectively. VAS (hand), VAS (foot), and FACT/GOG-NTX (NTX2, NTX4 and NTX8) were significantly correlated with PV. PV showed no correlation with a Disk-Criminator or the monofilament test used as a quantitative evaluation. The evaluation of CIPN is complex, and further improvement is required for evaluation with PV.

## Introduction

Chemotherapy-induced peripheral neuropathy (CIPN) is one of the most important adverse events that makes it difficult to continue chemotherapy^1–3^. There are numerous characteristics of CIPN such as burning/shooting pain, tingling, and numbness. There is little information on the relationship between numbness, tingling, and burning/shooting pain. Sensations of pain as reported by patients include “cold,” “burning,” and “dull,” or more descriptively as “walking on razor blades”^4^. The correlations between burning/shooting pain versus either numbness or tingling were weak^5^. Prevention and treatment of CIPN are indispensable in improving patient quality of life and promoting the continuation of chemotherapy. However, there are currently no effective precautions or treatments for CIPN^6,7^.

Electrophysiological examinations such as nerve conduction study (NCS) play a central role in the diagnosis of general peripheral neuropathy^8,9^. NCS is considered the gold standard for the objective evaluation method of diabetic polyneuropathy worldwide, but it is rarely applied to neuropathies caused by chemotherapy. NCS is useful in diagnosing neurological diseases by not only revealing the presence but also the severity classification of the diagnosis. However, measurement error is unavoidable, reproducibility is also considered a problem, and the abnormality rate of sensory nerve conduction velocity of diabetic neuropathy is not high^10^.

In the CIPN guidelines of the American Society Clinical Oncology (ASCO), 39 prevention clinical trials and six treatment clinical trials are cited^11^. In both cases, no diagnostic criteria for diagnosing the presence of CIPN is described, and only less than half of the patients have undergone electrophysiological examination and neurological examination. There is no clear criterion for those who undergo these tests, and it cannot be said that it is useful for judging the effect of existing diagnoses or treatment interventions. For the treatment and prevention of CIPN, an easy method to quantify CIPN is necessary.

The visual analogue scale (VAS) has been used in clinical and epidemiologic research to measure various symptoms^12^, including peripheral neuropathy due to diabetes^13,14^ and chemotherapy^15,16^. VAS was evaluated to determine the amount of pain perceived by the patients, who were explicitly asked to score the pain considered to be CIPN related. The reliability value was obtained for the VAS^17^. Pain measurement by VAS has an error of approximately ±20 mm^18^. Therefore, an assessment method with less error is also necessary to evaluate drugs to ameliorate PN.

The Pain Vision PS-2100 system (PV; Nipro Co., Osaka, Japan) was introduced clinically^19–23^. PV is an analytical instrument designed to evaluate patient sensory perception quantitatively. After measuring the Current Perception Threshold (CPT), the same method is used to measure the level of current that produces a sensation equivalent to pain. The stimulating current is generated after verifying that the patient can use the hand switch supplied with the kit. At the point the stimulating current is acknowledged as a sensation equivalent to pain, the hand switch button is pressed, finishing the measurement. Based on the CPT, the equivalent pain current is evaluated and can be displayed as pain degree values. The advantage of PV is that it can assess pain in a short time, as well as evaluate pain without causing pain to patients. However, the correlation between PV and other evaluation methods has not been reported. In this study, the correlation between assessment methods for CIPN was evaluated.

## Results

Between April 2014 and December 2015, a total of 73 patients received oxaliplatin chemotherapy for metastatic CRC. Registered patients included 37 men and 36 women in the range of 37 to 89 years (median age, 70 years). CIPN was evaluated a total of 483 times (median per patient six times) using VAS, FACT/GOG-NTX, Disk-Criminator, monofilament and PV methods. CIPN occurred in 73.9% of patients. PV could identify 78.1% of the symptoms of CIPN. The average VAS (hand), VAS (foot) and PV scores of CIPN were 18.4 (range: 0–100), 23.8 (range: 0–100) and 24.7 (range: 0–496), respectively. The average NTX1, NTX2, NTX3, NTX4, NTX5, NTX6, NTX7, NTX8, NTX9, HI12 and An6 were 0.9, 1.1, 0.8, 1, 0.5, 0.5, 0.3, 0.4, 0.3, 0.5 and 0.3, respectively (Table 1). The distribution of FACT/GOG-NTX, Disk-Criminator, and monofilament scores are as shown in Table 2.

**Table 1:** Baseline characteristics of patients.

	Average	SD	Min	5^th^ percentile	25^th^ percentile	Median	75^th^ percentile	95^th^ percentile	Max
Age	67.4	9.9	37	51	61	70	75	79	89
PainVision	24.7	46.5	0	0	1	8	28	103	496
VAS (hand)	18.4	27.4	0	0	0	6	24	89	100
VAS (foot)	23.8	29.9	0	0	0	10	44	88	100
NTX1	0.9	1	0	0	0	1	1	3	4
NTX2	1.1	1.1	0	0	0	1	2	3	4
NTX3	0.8	1	0	0	0	1	1	3	4
NTX4	1	1.1	0	0	0	1	2	3	4
NTX5	0.5	0.9	0	0	0	0	1	2	4
NTX6	0.5	1	0	0	0	0	1	3	4
NTX7	0.3	0.8	0	0	0	0	0	2	4
NTX8	0.4	0.8	0	0	0	0	1	2	4
NTX9	0.3	0.6	0	0	0	0	0	2	4
HI12	0.5	0.9	0	0	0	0	1	2	4
An6	0.3	0.7	0	0	0	0	0	2	4
FACT/GOG NTX									
Total score	6.7	7.2	0	0	1	4	10	20	37

SD: standard deviation; Min: minimum; Max: maximum; VAS: visual analogue scale; NTX1: numbness and tingling in the hands; NTX2: numbness and tingling in the feet; NTX3: discomfort in the hands; NTX4: discomfort in the feet; NTX5: joint pain/muscle cramps; NTX6: trouble hearing; NTX7: ringing/buzzing in the ears; NTX8: trouble buttoning buttons; NTX9: trouble feeling the shape of small objects; HI12: feeling weak all over; An6: trouble walking.

**Table 2:** The distribution of FACT/GOG-NTX, Disk-Criminator, and monofilament scores.

FACT/GOG-NTX	0	1	2	3	4			
NTX1	221	148	62	46	4			
NTX2	190	124	94	70	3			
NTX3	232	143	62	40	4			
NTX4	211	111	92	59	8			
NTX5	335	79	44	18	5			
NTX6	355	74	10	29	13			
NTX7	396	59	5	12	9			
NTX8	355	86	24	13	3			
NTX9	362	91	25	2	1			
HI12	322	99	39	14	7			
An6	371	69	30	8	2			
**Disk-Criminator (mm)**	**2**	**3**	**4**	**5**	**6**	**7**	**8**	**9**
	85	186	125	50	15	9	6	2
**Monofilament**	**Green**	**Blue**	**Purple**	**Red**				
	316	156	10	0				

FACT/GOG-NTX: Functional Assessment of Cancer Therapy/Gynecologic Oncology Group–Neurotoxicity.

**Figure 1:**
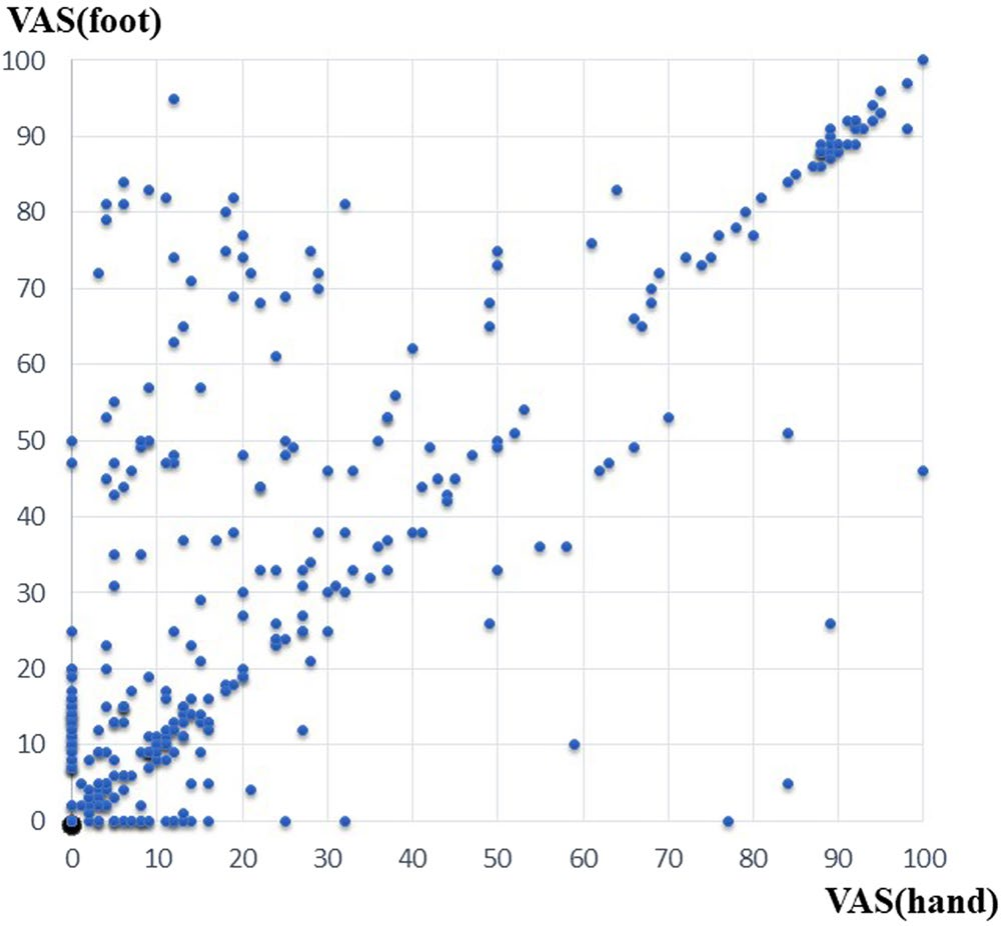
Correlation between VAS (hand) and VAS (foot) scores.

A strong positive correlation was found between VAS (hand) and VAS (foot) scores (r = 0.798) (Fig. 1). Each data point represents one assessment from a single patient. The average value obtained by subtracting the VAS (hand) from VAS (foot) was 5.38 (SD: 18.32), which was not significant in the t-test (p < 0.001). This finding indicates that the average VAS (foot) value was higher than the VAS (hand) value.

**Figure 2:**
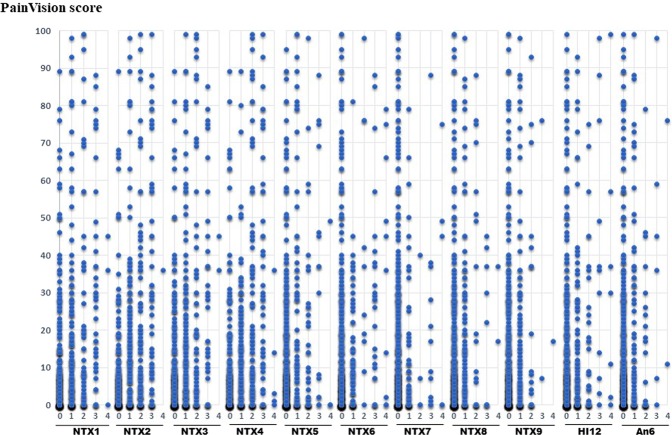
Distribution of PainVision and FACT/GOG-NTX scores.

**Figure 3:**
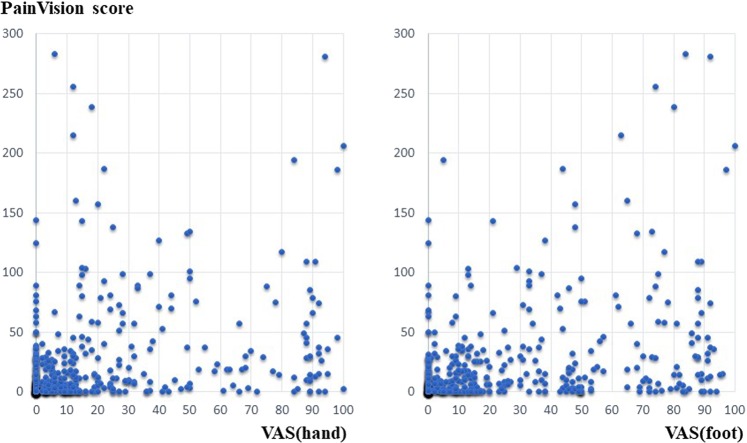
Correlation between PainVision and VAS (hand) or VAS (foot) scores.

**Figure 4:**
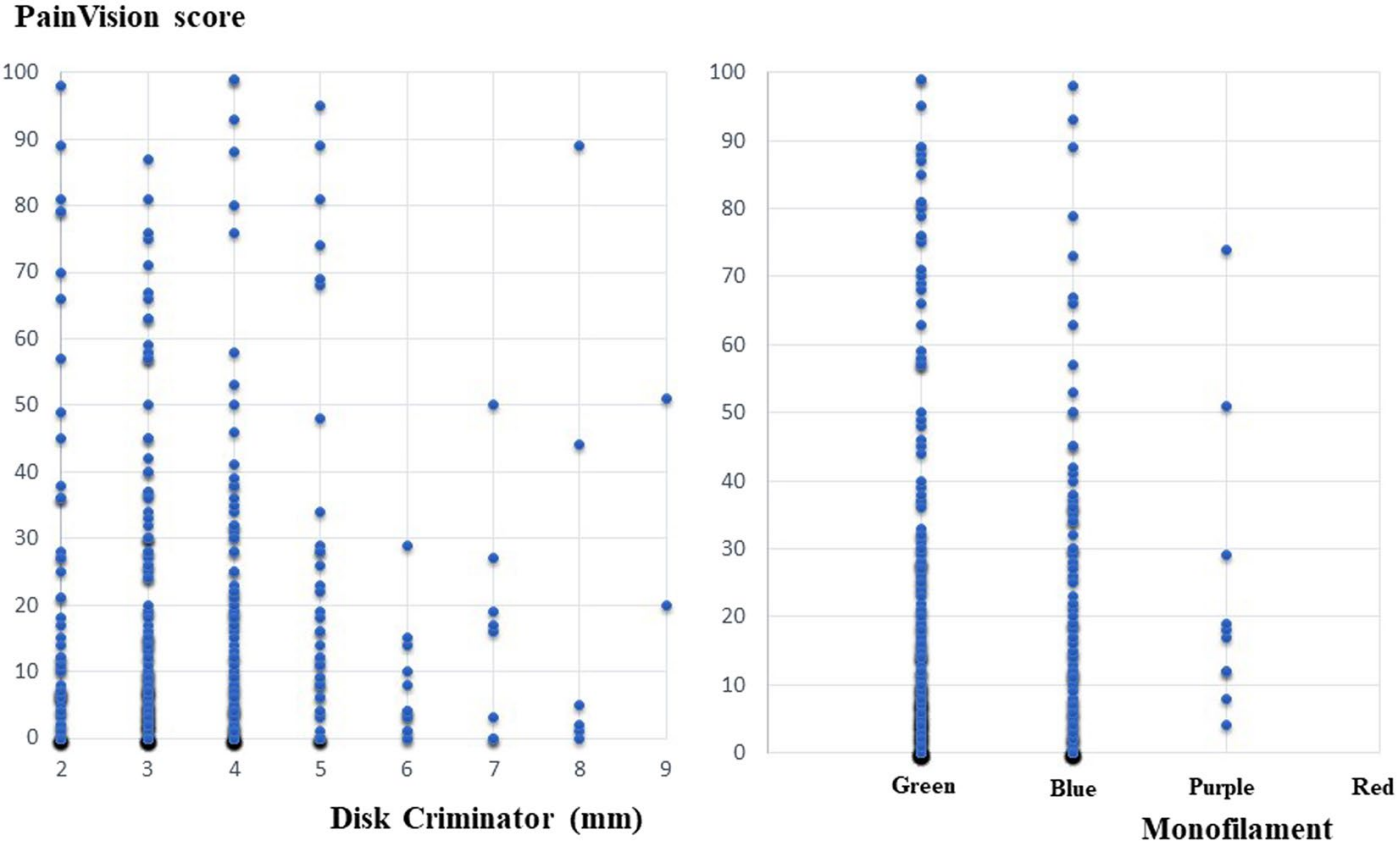
Distribution of PainVision and Disk-Criminator or monofilament scores.

**Figure 5:**
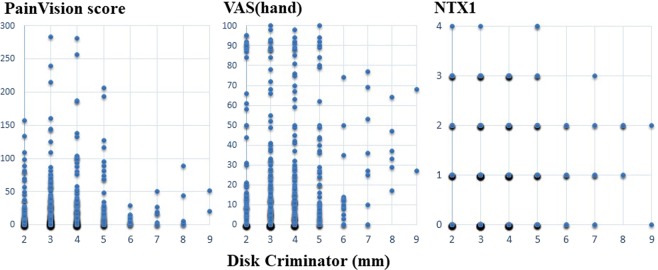
Correlation between Disk-Criminator and PainVision scores, with VAS (hand) or NTX1 scores.

**Figure 6:**
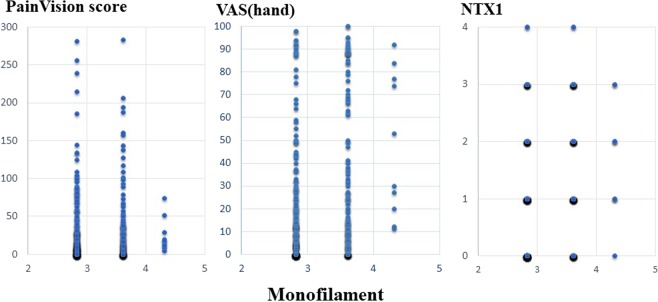
Correlation between monofilament and PainVision scores, with VAS (hand) or NTX1 scores.

A scatter plot of PV and FACT/GOG-NTX, a scatter plot of PV and VAS, a scatter plot of PV, and Disk-Criminator or monofilament are shown in Figs. 2, 3 and 4, respectively. In Fig. 2, the horizontal axis represents a score of 0–4 for each FACT/GOG-NTX question item, and the vertical axis represents the PV score. Despite evaluating the same symptoms, neither show a strong correlation. These figures do not take into account intraindividual variability. Thus, in Table 3, the results of hierarchical mixed models, including random individual effects and fixed effects of age and sex are shown. VAS (hand), VAS (foot), NTX 2, NTX 4 and NTX 8 were significantly associated with PV. There were no significant associations of the Disk-Criminator™ and monofilament methods with PV scores (Table 3). The associations between the repeated measurements of changes from the initial evaluations of VAS (hand), VAS (foot), Disk-Criminator, monofilament, and FACT/GOG-NTX, and those in PV were analyzed using a hierarchical mixed model to determine the best method for detecting increased CIPN symptoms when patients received additional chemotherapy (Table 4). The lowest P-value was VAS (foot), which correlated best with the amount of change over time. In Fig. 5, the lower the Disk-Criminator score, the higher the PV score, VAS (hand), and NTX1 values. Those with a Disk-Criminator score of 9 did not have high PV scores, VAS (hands), and NTX1 values. Similar findings were also observed for the monofilament score (Fig. 6).

**Table 3:** The associations of VAS (hand), VAS (foot), Disk-Criminator, monofilament, and FACT/GOG-NTX with Pain Vision score.

Dependent variable	Explanatory variable	Crude analysis			Adjusted for sex and age		
		Parameter estimate	Standard error	P value	Parameter estimate	Standard error	P value
VAS (hand)	PainVision	0.040	0.016	0.012	0.040	0.016	0.011
	Sex (F vs M)	—	—	—	−6.990	5.116	0.173
	Age	—	—	—	0.184	0.230	0.426
VAS (foot)	PainVision	0.067	0.019	0.001	0.067	0.019	0.001
	Sex (F vs M)	—	—	—	−0.104	5.412	0.985
	Age	—	—	—	0.106	0.246	0.665
Disk-Criminator	PainVision	0.002	0.001	0.145	0.002	0.001	0.146
	Sex (F vs M)	—	—	—	0.043	0.208	0.837
	Age	—	—	—	0.004	0.010	0.678
Monofilament	PainVision	0.001	0.001	0.308	0.001	0.001	0.307
	Sex (F vs M)	—	—	—	−0.105	0.081	0.195
	Age	—	—	—	0.016	0.004	<0.0001
NTX1	PainVision	0.001	0.001	0.164	0.001	0.001	0.153
	Sex (F vs M)	—	—	—	−0.363	0.200	0.070
	Age	—	—	—	0.005	0.009	0.571
NTX2	PainVision	0.003	0.001	0.001	0.003	0.001	0.001
	Sex (F vs M)	—	—	—	−0.051	0.217	0.814
	Age	—	—	—	0.007	0.010	0.490
NTX3	PainVision	0.001	0.001	0.095	0.001	0.001	0.096
	Sex (F vs M)	—	—	—	−0.181	0.199	0.365
	Age	—	—	—	0.000	0.009	0.979
NTX4	PainVision	0.003	0.001	0.001	0.003	0.001	0.001
	Sex (F vs M)	—	—	—	0.112	0.227	0.622
	Age	—	—	—	−0.002	0.010	0.853
NTX5	PainVision	0.000	0.001	0.478	0.000	0.001	0.494
	Sex (F vs M)	—	—	—	0.175	0.179	0.329
	Age	—	—	—	0.008	0.008	0.342
NTX6	PainVision	0.000	0.000	0.454	0.000	0.000	0.462
	Sex (F vs M)	—	—	—	−0.256	0.183	0.164
	Age	—	—	—	0.015	0.008	0.065
NTX7	PainVision	0.000	0.000	0.552	0.000	0.000	0.566
	Sex (F vs M)	—	—	—	−0.291	0.176	0.099
	Age	—	—	—	0.003	0.008	0.693
NTX8	PainVision	0.001	0.000	0.033	0.001	0.000	0.029
	Sex (F vs M)	—	—	—	−0.452	0.183	0.014
	Age	—	—	—	0.006	0.008	0.427
NTX9	PainVision	0.000	0.000	0.543	0.000	0.000	0.508
	Sex (F vs M)	—	—	—	−0.299	0.135	0.027
	Age	—	—	—	0.000	0.006	0.936
HI12	PainVision	0.000	0.001	0.525	0.000	0.001	0.531
	Sex (F vs M)	—	—	—	−0.052	0.187	0.782
	Age	—	—	—	−0.004	0.009	0.605
An6	PainVision	0.001	0.001	0.174	0.001	0.001	0.170
	Sex (F vs M)	—	—	—	−0.062	0.140	0.657
	Age	—	—	—	0.012	0.006	0.063

F: Female; M: Male.

**Table 4:** The associations between repeated measurements of the changes from the initial evaluations in VAS (hand), VAS (foot), Disk-Criminator, monofilament, and FACT/GOG-NTX, and those in PainVision.

Dependent variable	Explanatory variable	Crude analysis			Adjusted by gender and age		
		Parameter estimate	Standard error	P value	Parameter estimate	Standard error	P value
VAS (hand)	PainVision	0.02948	0.01491	0.0487	0.03044	0.01489	0.0415
	Sex (F vs M)				−6.6445	3.0899	0.0321
	Age				−0.00703	0.1434	0.9609
VAS (foot)	PainVision	0.0588	0.01824	0.0014	0.0609	0.0182	0.0009
	Sex (F vs M)				−6.6586	3.5669	0.0627
	Age				0.1557	0.1663	0.3497
Disk-Criminator™	PainVision	0.00211	0.001292	0.1033	0.002116	0.001297	0.1035
	Sex (F vs M)				−0.09614	0.2522	0.7032
	Age				−0.00262	0.01176	0.8237
Monofilament	PainVision	0.000714	0.000516	0.1669	0.00072	0.000517	0.1642
	Sex (F vs M)				−0.07772	0.1071	0.4684
	Age				−0.00091	0.00497	0.8546
NTX1	PainVision	0.000306	0.000627	0.6262	0.000323	0.000627	0.6072
	Sex (F vs M)				−0.2264	0.1401	0.107
	Age				−0.00236	0.006461	0.7148
NTX2	PainVision	0.002176	0.000673	0.0013	0.002212	0.000673	0.0011
	Sex (F vs M)				−0.219	0.1293	0.0911
	Age				0.000478	0.006032	0.9369
NTX3	PainVision	0.000502	0.000609	0.411	0.00055	0.000608	0.3661
	Sex (F vs M)				−0.3028	0.1464	0.0392
	Age				0.007758	0.006716	0.2487
NTX4	PainVision	0.001926	0.000705	0.0065	0.001996	0.000702	0.0047
	Sex (F vs M)				−0.3001	0.1448	0.0388
	Age				0.009799	0.006714	0.1453
NTX5	PainVision	−0.00009	0.000651	0.8918	−0.00003	0.00065	0.9624
	Sex (F vs M)				−0.1449	0.1296	0.2642
	Age				0.009592	0.006028	0.1124
NTX6	PainVision	−0.00093	0.000465	0.0463	−0.0009	0.000464	0.0532
	Sex (F vs M)				−0.04393	0.09463	0.6428
	Age				0.008277	0.004393	0.0603
NTX7	PainVision	−0.00092	0.000444	0.0385	−0.00089	0.000443	0.0442
	Sex (F vs M)				−0.09448	0.08852	0.2865
	Age				0.006984	0.004116	0.0905
NTX8	PainVision	0.000825	0.00045	0.0676	0.000832	0.000451	0.0656
	Sex (F vs M)				−0.1084	0.09838	0.2711
	Age				−0.00151	0.004544	0.7398
NTX9	PainVision	0.000105	0.000431	0.8086	0.000132	0.000431	0.7601
	Sex (F vs M)				−0.1131	0.08172	0.1673
	Age				0.002137	0.003816	0.5758
HI12	PainVision	−0.00016	0.000706	0.82	−0.00017	0.000708	0.8137
	Sex (F vs M)				0.06344	0.1599	0.6918
	Age				−0.00069	0.007369	0.9256
An6	PainVision	0.000112	0.000496	0.8214	0.000111	0.000498	0.824
	Sex (F vs M)				0.07782	0.08808	0.3775
	Age				0.001431	0.004133	0.7293

F: Female; M: Male.

## Discussion

VAS is one of the most common methods used to assess pain^24^. VAS is a method of grading pain currently experienced by patients compared with the worst imaginable pain^25^. Because of its ease of use, VAS has become a popular tool to quantify pain relief and pain intensity. VAS is an effective and reliable means to assess pain, depression, anxiety, and mood^24^. VAS tends to focus only on pain intensity, with an increased risk of over-simplification of the experience^26^.

Wang *et al*. showed a difference between the electrophysiological findings and the subjective signs reported by CIPN patients^27^. They also state that the severity of clinical sensory neuropathy does not always correlate with nerve conduction test findings. Conversely, Argyriou *et al*. reported that the nerve conduction test is useful for objectively evaluating the extent of CIPN, enabling the identification of asymptomatic peripheral neuropathy before onset^28^. In our study, VAS (hand), VAS (foot), NTX 2, NTX 4 and NTX 8 significantly correlated with PV in the analyses using a regression model with random effects of individual IDs adjusted by sex and age. Sex differences in the experience of clinically and experimentally induced pain are widely reported^29^, pain sensitivity is also thought to decrease with increasing age. However, as shown in Fig. 3, if intraindividual variability is not considered, the correlation coefficient between PV and VAS decreases. The Disk-Criminator and monofilament results did not associate with PV results. This discrepancy is because PV and VAS were used to assess pain, whereas the Disk-Criminator was used to measure spatial acuity in tactile sensations and monofilament was used to measure tactile sensitivity. Prior research has shown that numbness and tingling symptoms are correlated, but they are not necessarily correlated with pain^30^.

Several studies have analyzed the sensitivity to change over time in each of the proposed CIPN measures, in an attempt to test sensitivity to change over time^31–34^. Table 4 shows the association between repeated measures of changes from the initial evaluations of VAS (hand), VAS (foot), Disk-Criminator, monofilament, and FACT/GOG-NTX, and those in PV. This result suggested that VAS is the most representative measure of changes in neuropathy over time. In Table 3, there was a significant difference in NTX8, but as shown in Table 4, the significant difference disappeared. NTX8 indicates a response of “I have trouble buttoning buttons,” and we posit that the patient was able to learn the movement with time.

PV is used for the quantification of the intensity of pain. In clinical practice, this method is used not only for chronic pain such as fibromyalgia^35^ and lower back pain due to spondylolisthesis^36^ but also for acute pain caused by the removal of wound dressings^37^. Previous studies have shown that PV is a useful device that can quantitatively evaluate pain in various fields^38–40^. Patient-reported outcomes of CIPN related symptoms should always be included in clinical trials^41^. Measures of clinician-rated neuropathy signs and function measures are also encouraged. Although an evaluation method that can quantify CIPN enables interindividual and intraindividual comparison, Sato *et al*. reported that there was no significant difference between PV and CTCAE grades in the evaluation of CIPN^42^. If the severity of CIPN and PV do not correlate, PV cannot be used to evaluate clinical trials aimed at improving CIPN. Although PV significantly correlated with VAS (hand), VAS (foot), NTX2 and NTX4 (Table 3), it does not appear in Fig. 3 to be correlated. Therefore, it is necessary to make improvements that are also correlated visually. Because PV was developed for pain assessment, the following four conditions are considered as speculations to improve the evaluation of CIPN: (1) Measurement by simultaneous stimulation of multiple parts; (2) Machine body and software corresponding to increase and decrease of stimulus in one measurement; (3) the optimal stimulation wave; (4) the ease of input for stimulus detection. Clinical trials for the prevention and treatment of CIPN require the identification of optimal outcome measures to define the CIPN phenotype and the setting of parameters that lead to the evaluation of clinically relevant effects^43^. If these four conditions are satisfied, the correlation coefficient seems to rise further.

This study has a limitation. We should have reported the change in each CIPN measure vs. time and the associated factors. Recently, oxaliplatin has been stopped before CIPN has developed with the spread of the Stop & Go strategy^44^, and oxaliplatin has been reduced or suspended immediately after CIPN has developed. In addition, there are individual differences in the timing of CIPN. By these two points, we could not report change in each CIPN measure vs. time and the associated factors. The associations between the repeated measures of changes from the initial evaluations were investigated as an alternative (Table 4). To the best of our knowledge, there has been no previous study regarding the correlation between PV and other assessment in CIPN patients. We believe that the effect of the drug for CIPN should be evaluated quantitatively. Further research and effort are needed to improve the evaluation of CIPN by PV.

## Conclusions

Evaluation of CIPN is complex because numerous factors are involved. To apply quantitative evaluation methods to CIPN clinical trials, PV requires various improvements.

## Methods

### Study design

This study was approved by the Institutional Review Board of Fukuoka University Hospital (No. 13-4-7) and was performed between April 2014 and December 2015. Seventy-three patients with histologically confirmed metastatic colorectal adenocarcinoma, and treated with oxaliplatin as the first line of chemotherapy, were enrolled in the study. Patients exhibiting mental health issues that rendered the concepts of PV impossible to understand were excluded from this study. Patients who had peripheral neuropathy or musculoskeletal pain that could interfere with the measurement of quantitative pain before chemotherapy were also excluded. Informed consent was obtained from all patients before participation in this study. All methods were implemented according to the Declaration of Helsinki. This study included a different patient cohort than our previously reported studies^19,20,23^.

CIPN was defined using the National Cancer Institute Common Terminology for Adverse Events^45^. The measurement started from the second cycle and was performed before administration. Measurements are recorded continuously every 3 weeks in line with chemotherapy but are halted when chemotherapy is postponed because of adverse events. During treatment with oxaliplatin, measurements were recorded until the patient refused further measurement, and all measurements were analysed.

### VAS and the functional assessment of cancer therapy/Gynecologic Oncology Group - Neurotoxicity (Fact/GOG-NTX)

**Figure 7:**
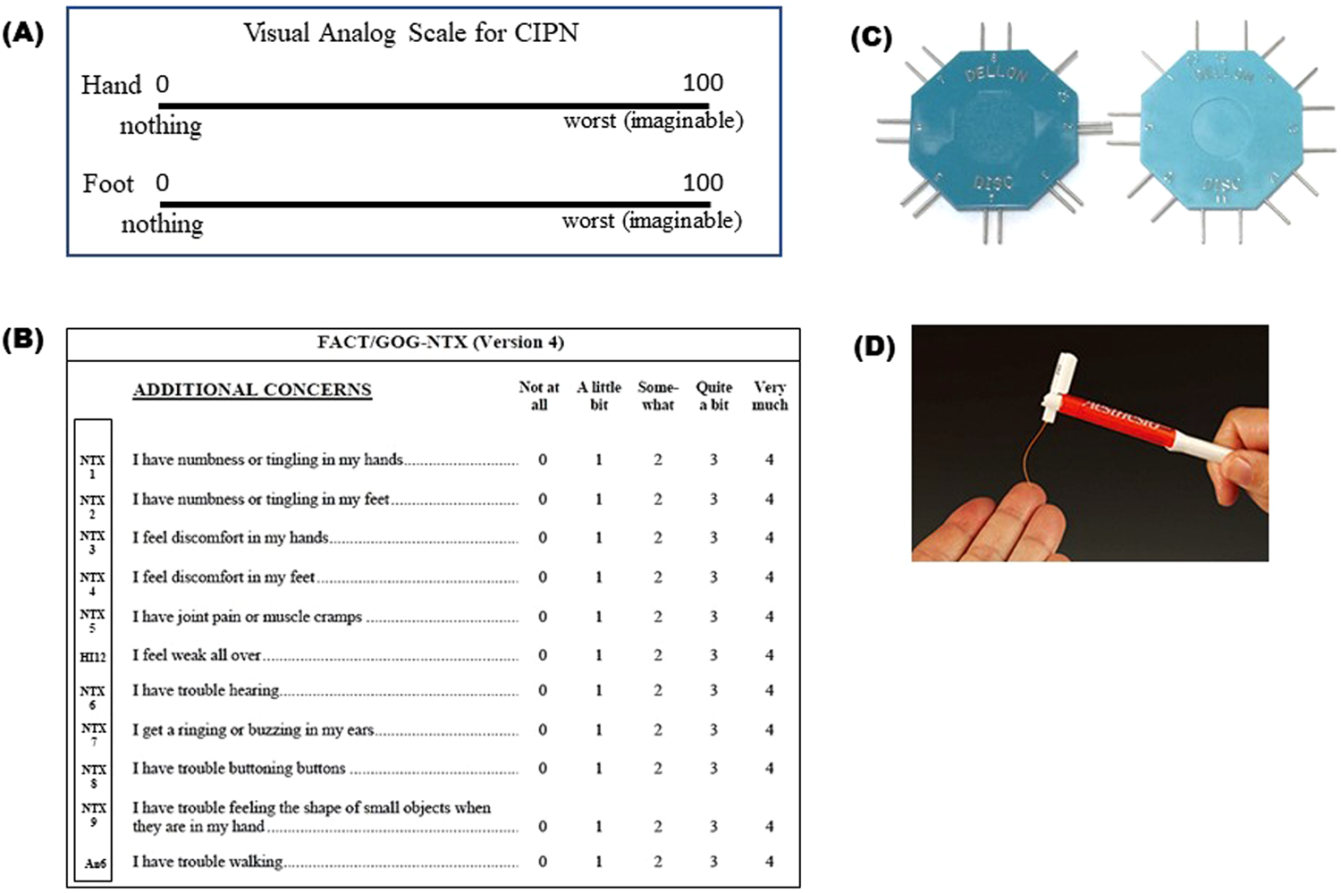
Subjective and objective evaluation methods used in this study. (**A**) VAS, (**B**) FACT/GOG-NTX, (**C**) Disk-Criminator, (**D**) Monofilament.

VAS is a commonly used method for assessing the fluctuation of pain intensity. Patients are instructed to indicate the perceived pain intensity by marking on a 100-mm horizontal line labelled “0 (no pain)” at the left end and “100 (worst imaginable pain)” on the right end (Fig. 7A). VAS was used to assess chronic CIPN subjectively before each cycle of chemotherapy. The patient was instructed to consider only neuropathic pain present on the day of the measurement.

The FACT/GOG-NTX is an 11-item subscale for evaluating symptoms associated with chemotherapy-induced neuropathy (Fig. 7B). We examined the correlation between these 11 items and PV.

### PainVision PS-2100

PV was developed as a medical device that evaluates the strength of pain using a numerical value^46^. The measurement principle of the system is to compare a unique electrical stimulation with the pain experienced by the patient^19,37^. A painless electrical stimulation whose intensity is equivalent to the intensity of the pain experienced by the patient is applied, and the current value of this electrical stimulation is defined as “pain-compatible electrical current.” The patient’s threshold for the electrical stimulation is defined as the “current perception threshold” which is intended to eliminate inter-individual variability. With these two values, pain intensity is defined by the following equation:

$$\begin{array}{c}{\rm{Pain}}\,{\rm{intensity}}=({\rm{pain}} \mbox{-} {\rm{compatible}}\,{\rm{electrical}}\,{\rm{current}}-{\rm{current}}\,{\rm{perception}}\,{\rm{threshold}})/\\ \,{\rm{current}}\,{\rm{perception}}\,{\rm{threshold}}\times 100.\end{array}$$

An electrode is mounted on the inside surface of the forearm. An electrical current is made to flow (50 Hz; 0–150 µA RMS; pulse width: 0.3 ms), and the stimulation is strengthened^19,22^. The patient is instructed to press a button the first time she/he perceives this stimulus; the current at this point is defined as the “minimum perceived current” value. As the stimulation current is increased, the patient is instructed to press the switch when they feel that the intensity of the stimulation current is equivalent to the intensity of the pain they are experiencing. The current is defined at this point as the “pain-equivalent current” value. Using the obtained values, “pain intensity” is calculated using the above formula. In the absence of pain, the value is 0 and increases according to the degree of pain. There is no upper limit. Each measurement is easily completed in a few minutes. PV was used to assess symptoms related to chronic CIPN subjectively before each cycle of chemotherapy.

### The Disk-Criminator test

The Disk-Criminator is a two-point discrimination (TPD) measuring device^47^ (Fig. 7C). The TPD method is a method that is completed in a shorter time than the nerve conduction test, is less painful, practical, cost-effective, and more easily applicable^48^. The method was performed as previously reported^49^. The Disk-Criminator has nine levels of discrimination, the first being 0 mm, or 1 point, whereas the rest were 2 points, with distances between the 2 points of 1 mm to 9 mm. For 2-point discrimination testing, patients were asked to respond with the number (1 or 2) they felt most accurately indicated the stimulus. The patient was blinded, and the hand immobilized. The tester applied just enough pressure to depress the ventral side of index finger directly below the instrument, and the points contacted the skin simultaneously. The placement of 1 or 2 points was randomly mixed. Each subject was assessed three times on each of the nine distances on the Disk-Criminator. The number of correct responses was the 2-point discrimination score. Measurements were taken directly before each treatment cycle.

### The monofilament test

The monofilament test is an easy-to-use, inexpensive, and portable test for evaluating the loss of protective sensation and is recommended by several practical guidelines to detect peripheral neuropathy^50,51^. The test was performed using a Semmes-Weinstein aesthesiometer (Research Design, Inc., Houston, TX, USA) (Fig. 7D). The filament contacts the ventral side of the patient’s index finger. With a loss of sensation, the patient cannot detect the presence of the filament. The higher the value of the monofilament, the stiffer and harder it is to bend. Four monofilaments used to diagnose peripheral neuropathy are the 2.83/0.07 g (Green), 3.61/0.4 g (Blue), 4.31/2.0 g (Purple) and 4.56/4.0 g (Red). The monofilaments were applied slowly and precisely to the skin of the finger in the same fashion for the same amount of time for each test. It was pressed to produce a slight bend. Every trial involved touching the patient and then recording whether the patient reported, “Yes, I was touched” or “No, I was not touched.” Each subject was assessed three times with each of the monofilaments. The smallest monofilament color that the patient could feel was entered. Measurements were taken directly before each treatment cycle.

### Statistical analyses

Data were analysed using SAS Version 9.4 (SAS Institute, Cary, North Carolina, USA). To investigate the reliability of the device, the quantified pain degree score was evaluated twice. Each measurement was performed twice, and the average value was used. Data are presented as the mean ± standard deviation (SD), median (interquartile range 25–75%), or the number of participants (percentages). The associations of VAS (hand), VAS (foot), Disk-Criminator, monofilament, and FACT/GOG-NTX with PV were assessed using hierarchical mixed models including random individual effects with or without fixed effects of age and sex. The associations between the repeated measures of changes from the initial evaluations in VAS (hand), VAS (foot), Disk-Criminator, monofilament, and FACT/GOG-NTX, and those in PV were evaluated using a hierarchical mixed model. P values less than 0.05 were considered statistically significant.

## Data Availability

The data that support the findings of this study are available from the corresponding author upon reasonable request.
